# Bronchial artery embolization for the management of frequent hemoptysis caused by bronchiectasis

**DOI:** 10.1186/s12890-022-02198-2

**Published:** 2022-11-01

**Authors:** Guang-Dong Lu, Hai-Tao Yan, Jin-Xing Zhang, Sheng Liu, Hai-Bin Shi, Qing-Quan Zu

**Affiliations:** Department of Interventional Radiology, the First Affiliated Hospital with Nanjing Medical University, 300 Guangzhou Road, Gulou District, Nanjing, 210029 Jiangsu China

**Keywords:** Bronchial arteries, Bronchiectasis, Embolization, Therapeutic, Hemoptysis

## Abstract

**Background:**

To retrospectively evaluate the effectiveness of bronchial artery embolization (BAE) compared with conservative therapy for the treatment of frequent hemoptysis caused by bronchiectasis.

**Methods:**

From January 2015 to December 2019, consecutive patients who were admitted due to frequent (more than three times per year) bronchiectasis-related hemoptysis were retrospectively reviewed. Those who were treated with either BAE (*n* = 69) or conservative therapy (*n* = 47) were enrolled for analysis. The technical success, clinical success, and complications of the BAE procedure were evaluated. Long-term hemoptysis-free survival rates and clinical success were compared between patients in the BAE group and patients in the conservative group. A Cox proportional hazard regression model was used to identify the predictors of recurrent hemoptysis.

**Results:**

The technical success rate was 100% for the BAE procedure, and clinical success was achieved in 92.8% (64 of 69) of cases. No major procedure-related complications occurred, and minor complications were observed in 16 cases (23.2%). The 1-, 2-, and 3-year hemoptysis-free survival rates were 88.3, 71.3, and 66.2%, respectively, for the BAE group and 31.9, 17.6, and 2.5%, respectively, for the conservative treatment group (*P* <  0.001). Multivariate analysis showed that BAE was a protective factor against recurrent hemoptysis in treated patients. In addition, the presence of cystic bronchiectasis was the only independent risk factor for rebleeding in the whole population and in the BAE group.

**Conclusions:**

BAE may provide an effective option for patients with frequent bronchiectasis-related hemoptysis, especially for those without cystic bronchiectasis.

**Supplementary Information:**

The online version contains supplementary material available at 10.1186/s12890-022-02198-2.

## Background

Bronchiectasis is a chronic respiratory disease characterized by repeated airway infections, enlargement of bronchi, productive cough, and recurrent exacerbations [1–3]. There has been growing interest in this disease over the past decade due to its increasing prevalence, associated mortality and significant financial burden [2, 4, 5]. Due to the structural damage caused by recurrent airway inflammation, 26.0–51.2% of these patients are estimated to have hemoptysis [6–8]. The occurrence of frequent episodes of hemoptysis accompanying a cascade of acute exacerbations can eventually lead to increased mortality and decreased quality of life [9–11]. A recent study also confirmed that hemoptysis is positively correlated with the incidence of depression in bronchiectasis patients [12].

Treatment for frequent bronchiectasis-related hemoptysis is still an open question in clinical practice. Prescription of a hemostatic drug always results in a relatively short-term hemoptysis-free period. Lobectomy or segmentectomy can provide a definite curative effect but is limited to patients with sufficient respiratory reserve and localized lesions [13, 14]. Currently, the bronchial artery embolization (BAE) procedure, which has a stable hemostatic rate, has been accepted as the first-line treatment for life-threatening hemoptysis [15–17]. The immediate hemostatic effect of BAE for massive hemoptysis caused by bronchiectasis has been well established in the literature [15, 18–20]. However, there is a lack of effective guidelines and active treatment for this kind of frequent hemoptysis that can cause anxiety and decrease quality of life.

We therefore performed this retrospective study to evaluate the efficacy of BAE compared with that of conservative therapy for the treatment of frequent bronchiectasis-related hemoptysis. We also tried to investigate factors associated with recurrence after successful hemostasis.

## Methods

### Study design

This study was a single tertiary referral center retrospective cohort analysis and was approved by the Ethics Committee of the First Affiliated Hospital of Nanjing Medical University. The requirement for informed consent was waived due to its retrospective nature. The study protocol followed the guidelines of the World Medical Association Declaration of Helsinki.

### Patients

**Fig. 1:**
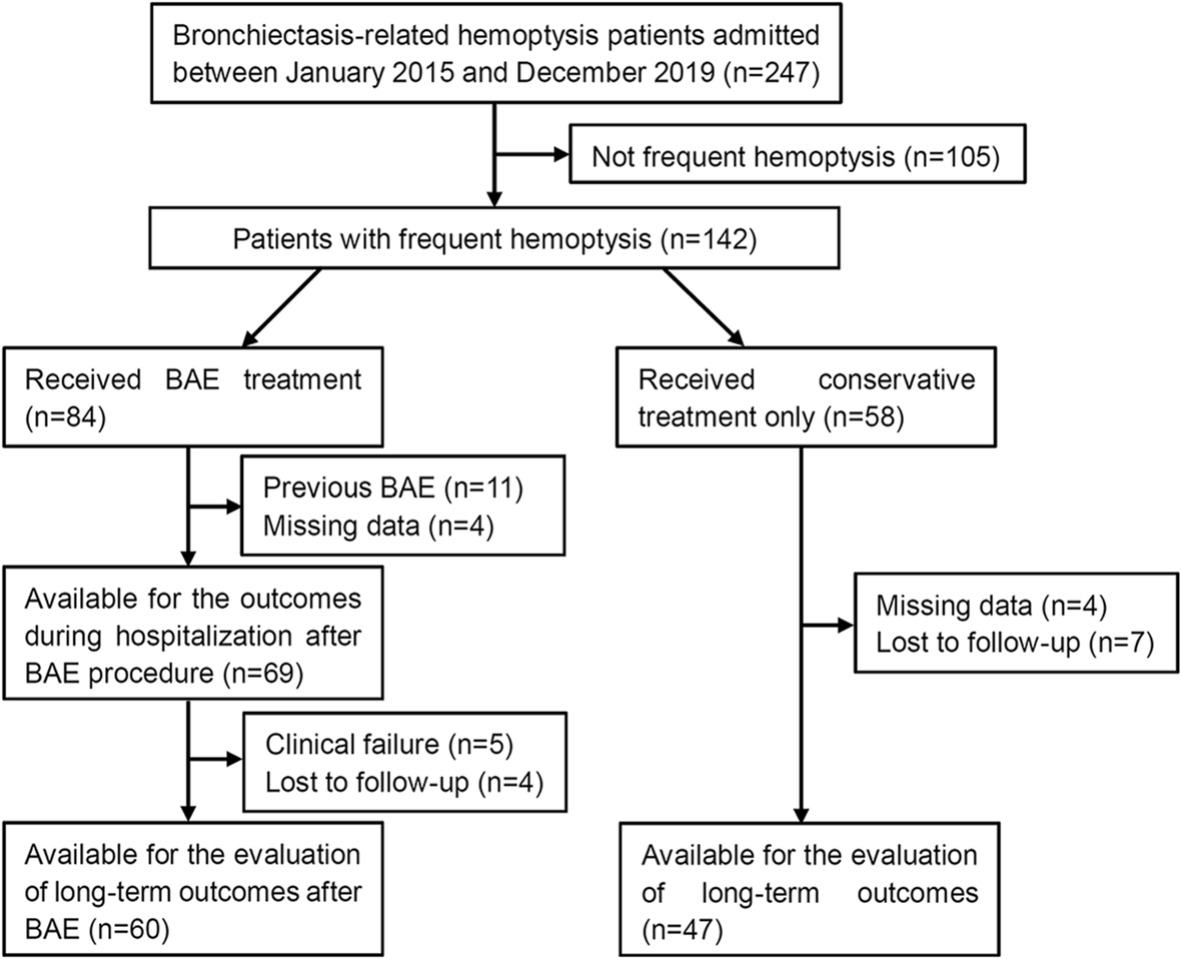
Flowchart of the included patients

In total, 247 consecutive patients with bronchiectasis-related hemoptysis were confirmed in our center between January 2015 and December 2019. The diagnosis of bronchiectasis was confirmed by high-resolution computed tomography with a ratio of the cross-sectional diameter of the inner airway and its concomitant artery (inner airway-artery ratio) > 1.0 [21]. Patients with frequent hemoptysis (defined as a history of hemoptysis of more than 1 year, at least three occurrences per year and a volume of hemoptysis of more than 20 ml each time) were reviewed for analysis. According to the treatment received during hospitalization, they were divided into a BAE group and a conservative treatment group. The flowchart of patients included is shown in Fig. 1. Ultimately, 69 patients were assessed for technique and clinical notes in the BAE group, while 47 patients were enrolled in the conservative treatment group. Noncontrast chest CT with or without CT angiography (CTA) was employed to evaluate the location, extent, and severity of bronchiectasis, the possible bleeding focus, and the suspicious culprit vessels. Features indicating bleeding sites on chest CT included local blood accumulation or inflammation in lung tissue, lung parenchymal destruction, bronchiectatic changes, hypertrophic tortuous bronchial or nonbronchial arteries, neovascularity, hypervascularity, contrast extravasation, bronchial artery aneurysm, pleural thickening and so on [20].

### Conservative therapy

After admission, standard medical management, including vital sign monitoring, hypoxemia correction, stabilization of blood pressure, hemostasis and anti-infection, was performed. Hemostatic drugs, e.g., pituitrin and phentolamine, were pumped continuously and continued until 3 days after the hemoptysis stopped. The combination of third-generation cephalosporins and quinolones was initially used as an empirical antibiotic and was adjusted during hospitalization according to culture results. Blood products were transfused as necessary according to the results of laboratory tests. In cases of massive hemoptysis, the patients received early endotracheal intubation in an intensive care setting. After discharge, patients were instructed to abandon smoking, reduce exposure to polluted air, receive respiratory rehabilitation, and take oral low-dose macrolides to control pulmonary infections.

### BAE procedures

All BAE procedures were performed by two interventional radiologists with over 6 years of experience. Access was achieved by a 5 F vascular sheath via the femoral artery under local anesthesia, and selective angiograms of the bronchial arteries and/or nonbronchial systemic collateral arteries (NBSAs) were performed with angiographic catheters (Cobra catheter, Rosch left gastric catheter or Mikaelsson catheter, Cook, USA). Positive angiographic findings included extravasation of the contrast agent, arterial hypertrophy and tortuosity, neovascularity, hypervascularity, systemic arterial-pulmonary circulation shunts, and aneurysms. These culprit arteries were embolized with polyvinyl alcohol (PVA) particles (300–500 μm; Cook, USA) or Embosphere particles (300–500 μm, 500–700 μm, Merit Maestro, USA) combined with gelatin sponge particles (350–560 μm; Hangzhou Alicon Pharmaceutical Co., Ltd., China). To avoid embolization of the important side branches or reflux of the embolic material, a microcatheter (2.7 F; Terumo, Japan; or 2.4 F; Merit Maestro, USA) was employed for embolization in every case. Procedure-related complications that resulted in prolonged hospitalization, advanced care, permanent sequelae, or death were regarded as major complications.

### Data collection and outcome evaluation

We collected data related to baseline clinical characteristics and noncontrast CT image features. Baseline clinical characteristics included age, sex, body mass index (BMI), history of hemoptysis, hemoptysis volume, smoking, comorbidities, admission systolic pressure, laboratory indexes and in-hospital days. The amount of hemoptysis per day was divided into three levels: mild (< 100 ml), moderate (100–300 ml), and massive (≥300 ml) [17].

**Fig. 2:**
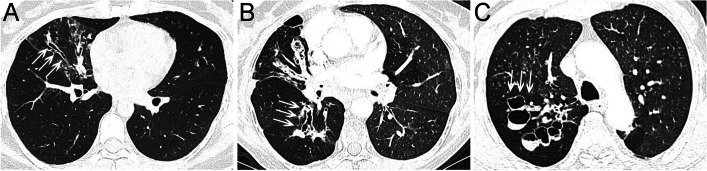
Computed tomography images of the three types of bronchiectasis. **A** Columnar bronchiectasis (white arrows); **B** Varicose bronchiectasis (white arrows); **C** Cystic bronchiectasis (white arrows)

Noncontrast CT image features were independently evaluated by two thoracic radiologists, and any differences reached a consensus by a third radiologist. The CT grading system was a modified version of that described by Reiff et al. [22]. Each lung segment was scored for (1) presence of bronchiectasis (0 = none, 1 = presence); (2) severity of bronchial dilatation (0 = normal, 1 = less than twice the diameter of the adjacent pulmonary artery, 2 = 2-3x the diameter of the adjacent pulmonary artery, 3 = more than 3x the diameter of the adjacent pulmonary artery); (3) severity of bronchial wall thickening (0 = normal, 1 = 0.5x the diameter of the adjacent pulmonary artery, 2 = 0.5-1x the diameter of the adjacent pulmonary artery, 3 = more than 1x the diameter of the adjacent pulmonary artery). The extent of bronchiectasis was taken as the sum of the scores for each of the lung segments. The severity of the bronchial dilatation score was calculated as the sum of the dilatation score for each segment divided by the total extent score, and the severity of the bronchial wall thickening score was estimated as the sum of the thickness score for each segment divided by the total extent score. The type of bronchiectasis was described as cylindrical, varicose, or cystic according to the Reid classification [23]. Representative images of these three bronchiectasis types are shown in Fig. 2. The diameter of the abnormal bronchial arteries was measured 1 cm from the aortic origin and perpendicular to the vessel axis on the angiographic images in the BAE group [24].

After discharge, the patients’ status was followed-up through outpatient or telephone visits in the first month after BAE and every 6 months thereafter. Clinical success was defined as no more coughing up of fresh blood during hospitalization. Recurrence was defined as a hemoptysis volume ≥ 30 ml/d, requirement for repeat BAE, requirement for lobectomy, or death due to recurrence. Recurrence-free time was defined as the duration between the date of hemostasis during this hospitalization to the date of recurrence or the date of last follow-up (October 2020 for available patients).

### Statistical analysis

Continuous variables are described as the mean ± standard deviation or median with interquartile range. Categorical variables between groups were compared with the χ^2^ test or Fisher’s exact test, while continuous variables were compared with the t test or Wilcoxon test. The recurrence-free survival rates were estimated by the Kaplan–Meier method, and the log-rank test was used to identify the differences between recurrence-free rate curves. Patients who died for reasons not related to hemoptysis were censored at the time of death. We examined predictive factors of recurrent hemoptysis by using univariate and multivariate Cox proportional hazards regression models, and factors with a *P* value < 0.05 in the univariate analysis were included in the multivariate analysis with the Enter method. Data analyses were performed with SPSS (version 24.0, Armonk, NY, USA), and *P* <  0.05 was considered statistically significant.

## Results

### Characteristics of BAE and short-term outcomes

For the 69 patients treated with BAE, technical success was achieved in all cases. Clinical success was achieved in 64 patients (92.8%), while four of them were lost to follow-up. Of the five patients without clinical success after BAE, two received conservative treatment, two underwent lobectomy, and one died because of uncontrolled hemoptysis.

A total of 181 arteries were embolized during the procedure: 162 bronchial arteries (90 right, 72 left) and 19 NBSAs (five intercostal arteries, three internal thoracic arteries, seven phrenic arteries, three esophageal arteries, and one thyrocervical trunk). The average number of culprit vessels embolized was 2.6 ± 1.2 arteries per patient. The average diameter of the bronchial arteries measured on the angiographic images was 3.1 mm (range, 1.9–6.3 mm).

Minor complications were observed in 23.2% (16 of 69) of the patients, including chest or back pain in 10 (14.5%), fever in 8 (11.6%), and puncture site hematoma in 2 (2.9%) patients. All these symptoms were resolved with medical care. No major procedure-related complications occurred.

### Baseline characteristics in the two groups

Raw data of the 107 patients with long-term outcomes is shown in Supplementary material 1 as an excel table. The baseline characteristics of them are shown in Table 1. The BAE group had more male patients, more severe hemoptysis, and higher white blood cell (WBC) counts at admission. The bronchiectasis types between groups were also different. The other variables were not significantly different between groups.

**Table 1:** The baseline characteristics of 107 patients with long-term outcomes

Variables	BAE (***n*** = 60)	Conservative (***n*** = 47)	***P*** value
Female, No. (%)	27 (45.0)	33 (70.2)	0.009
Age, years	59.88 ± 10.35	58.06 ± 10.63	0.347
BMI, kg/m^2^	22.27 ± 3.28	22.86 ± 3.03	0.463
History of hemoptysis ≥10 years, No. (%)	31 (51.7)	20 (42.6)	0.349
Extent of hemoptysis, No. (%)			< 0.001
< 100 mL	19 (31.7)	34 (72.3)	< 0.001
100–300 mL	26 (43.3)	11 (23.4)	0.031
≥ 300 mL	15 (25.0)	2 (4.3)	0.004
Smoke, No. (%)	12 (20.0)	7 (14.9)	0.493
Hypertension, No. (%)	13 (21.7)	10 (21.3)	0.961
Diabetes, No. (%)	4 (6.7)	2 (4.3)	0.693
Admission SBP, mmHg	124 (115–130)	128 (117–138)	0.464
Hemoglobin, g/L	123.03 ± 18.78	125.60 ± 16.58	0.278
PLT, *10^9^/L	185.75 ± 59.64	189.53 ± 51.81	0.770
WBC, *10^9^/L	7.97 ± 2.73	6.46 ± 1.91	0.002
Number of lung segments involved	4 (2–7)	5 (2–8)	0.578
Bronchial dilatation severity score	1.8 (1.0–2.7)	2.1 (1.7–2.6)	0.072
Bronchial wall thickening severity score	2.4 (1.8–3.0)	2.4 (2.0–3.0)	0.765
Bronchiectasis type, No. (%)			0.019
Columnar	17 (28.3)	5 (10.6)	0.025
Varicose	21 (35.0)	13 (27.7)	0.418
Cystic	22 (36.7)	29 (61.7)	0.01
In-hospital days	5 (4.0–7.8)	7 (6.0–9.0)	0.003
Hemoptysis recurrence, No. (%)	21 (35.0)	45 (95.7)	< 0.001
Follow-up period, months	31.2 (21.4–55.5)	27.7 (8.1–48.5)	–

Continuous data are presented as the mean ± standard deviation or median (interquartile range). *BAE* Bronchial artery embolization, *BMI* Body mass index, *SBP* Systolic blood pressure, *PLT* Blood platelet, *WBC* White blood cell

### Hemoptysis recurrence

**Fig. 3:**
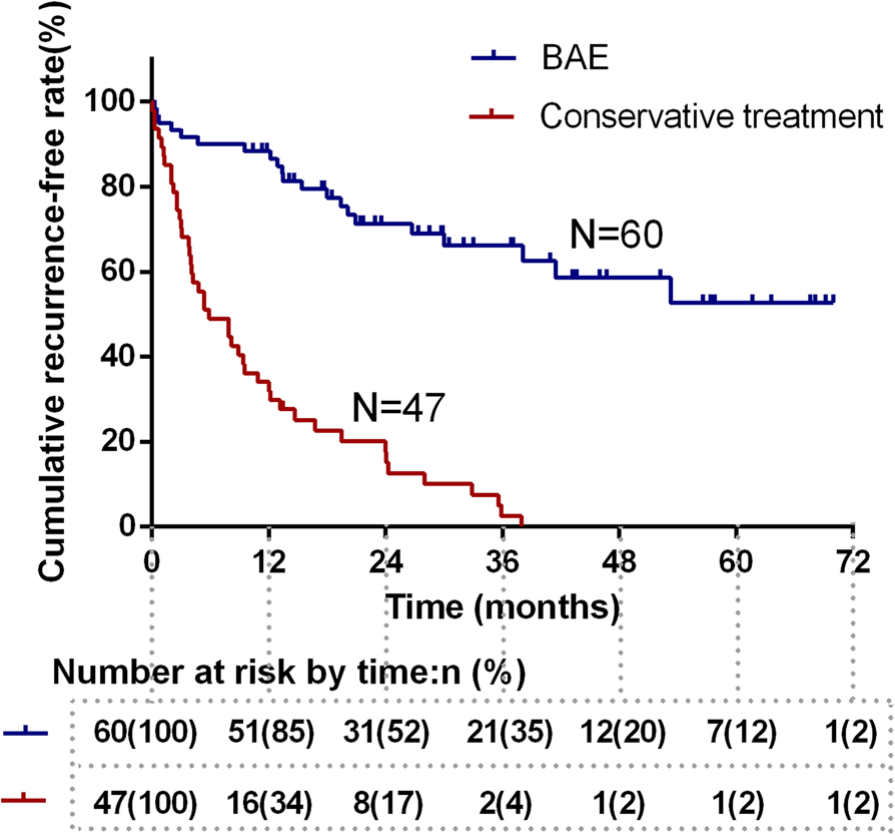
Cumulative recurrence-free rate curves stratified according to the treatment method used. The 1-, 2-, and 3-year hemoptysis-free survival rates were 88.3, 71.3, and 66.2%, respectively, for the BAE group and 31.9, 17.6, and 2.5%, respectively, for the conservative treatment group (*P* < 0.001)

During follow-up, recurrence of hemoptysis occurred in a total of 66 (66/107, 61.7%) patients (21 in the BAE group, 45 in the conservative group), including five patients in the conservative group who died directly related to recurrent hemoptysis. BAE was performed in twelve of these patients, lobectomy was performed in five patients, and conservative treatment was received by the others. The 1-, 2-, and 3-year hemoptysis-free survival rates were 88.3, 71.3, and 66.2% for the BAE group and 31.9, 17.6, and 2.5% for the conservative treatment group, respectively (*P* <  0.001) (Fig. 3).

**Fig. 4:**
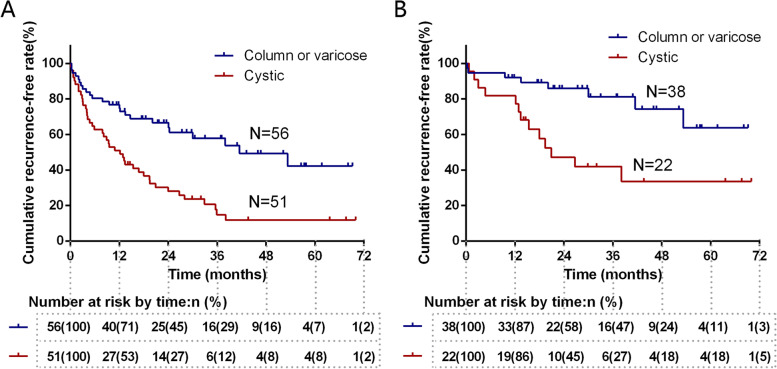
The estimated cumulative recurrence-free rate curves for patients with or without cystic bronchiectasis. **A** Recurrence-free rate curves for all 107 included patients [hazard ratio (HR), 2.63; 95% confidence interval (CI): 1.59–4.37; *P* < 0.001 by using Cox proportional regression analysis). **B** Recurrence-free rate curves for 60 patients who received BAE treatment (HR, 3.48; 95% CI: 1.44–8.43; *P* = 0.006)

Univariate and multivariate analyses of the variables associated with recurrence are shown in Table 2. Multivariate analyses demonstrated that the BAE procedure [hazard ratio (HR), 0.18; 95% confidence interval (CI), 0.10–0.33; *P* <  0.001] and bronchiectasis subtype (the level of cystic) (HR, 1.740; 95% CI, 1.03–2.94; *P* = 0.039) were independently associated with recurrence. The factors related to recurrent hemoptysis in the BAE group are shown in Table 3. Ultimately, only the bronchiectasis subtype of cystic (HR, 2.787; 95% CI, 1.117–6.956; *P* = 0.028) was an independent predictor for recurrent hemoptysis. The Kaplan–Meier estimated curves of recurrence-free survival for patients with or without cystic bronchiectasis in all patients and BAE subgroups are shown in Fig. 4.

**Table 2:** Univariate and multivariate analyses of the variables associated with recurrence of hemoptysis in all patients (*n* = 107)

Variables	Recurrence (*n* = 66)	Nonrecurrence (*n* = 41)	Univariate		Multivariate	
			HR (95% CI)	*P* value	HR (95% CI)	*P* value
Age, years	58.7 ± 10.2	59.8 ± 10.9	0.99 (0.96–1.01)	0.332		
Gender						
Female	42	18	1	–		
Male	24	23	0.65 (0.40–1.08)	0.098		
BMI, kg/m^2^	22.4 ± 3.0	22.8 ± 3.4	0.96 (0.89–1.04)	0.280		
History of hemoptysis ≥10 years	33	18	1.22 (0.75–1.98)	0.433		
Extent of hemoptysis, mL				0.069		
< 100	37	16	1	**–**		
100–300	22	15	0.85 (0.50–1.44)	0.550		
≥ 300	7	10	0.38 (0.17–0.87)	0.021		
Smoking	10	9	0.67 (0.34–1.31)	0.240		
Hypertension	14	9	1.00 (0.55–1.81)	0.996		
Diabetes	3	3	1.01 (0.32–3.24)	0.986		
Admission SBP, mmHg	126 (117, 139)	125 (114, 130)	1.01 (0.99–1.03)	0.118		
Admission hemoglobin (g/L)	123.6 ± 16.1	125.1 ± 20.5	1.00 (0.99–1.01)	0.808		
PLT, *10^9^/L	181.3 ± 56.4	197.3 ± 54.9	1.00 (0.99–1.00)	0.405		
WBC, *10^9^/L	6.7 ± 2.4	8.2 ± 2.4	0.87 (0.78–0.96)	0.009	0.95 (0.85–1.07)	0.412
Number of lobes involved	5 (2, 8.3)	4 (2, 7)	1.05 (0.99–1.12)	0.114		
Bronchial dilatation severity score	2.1 (1.7, 2.7)	1.8 (1.0, 2.6)	1.28 (0.94–1.75)	0.123		
Bronchial wall thickening severity score	2.4 (2.0, 3.0)	2.3 (1.7, 3.0)	1.10 (0.79–1.54)	0.582		
Bronchiectasis type						
Columnar or varicose	24	32	1	**–**	1	
Cystic	42	9	2.63 (1.59–4.37)	< 0.001	1.74 (1.03–2.94)	0.039
Treatment method						
BAE	21	39	0.14 (0.08–0.25)	< 0.001	0.18 (0.10–0.33)	< 0.001
Conservative therapy	45	2	1	–	1	
In-hospital days	7 (5, 8.3)	5 (4, 8.5)	1.02 (0.95–1.08)	0.624		

Continuous data are presented as the mean ± standard deviation or median (interquartile range). *HR* Hazard ratio, *CI* Confidence interval, *BMI* Body mass index, *SBP* Systolic blood pressure, *PLT* Blood platelet, *WBC* White blood cell, *BAE* Bronchial artery embolization

**Table 3:** Univariate and multivariate analyses of the variables associated with recurrence of hemoptysis in the patients treated with BAE (*n* = 60)

Variables	Recurrence (*n* = 21)	Nonrecurrence (*n* = 39)	Univariate		Multivariate	
			HR (95% CI)	*P* value	HR (95% CI)	*P* value
Age, years	58.7 ± 10.3	60.5 ± 10.5	0.98 (0.94–1.03)	0.437		
Gender						
Female	10	17	1	–		
Male	11	22	0.90 (0.38–2.11)	0.802		
BMI, kg/m^2^	21.2 ± 2.6	22.9 ± 3.5	0.84 (0.72–0.97)	0.017	0.87 (0.75–1.02)	0.085
History of hemoptysis ≥10 years	14	17	2.56 (1.02–6.41)	0.046	2.13 (0.82–5.52)	0.120
Extent of hemoptysis, mL				0.295		
< 100	5	14				
100–300	11	15	2.20 (0.76–6.35)	0.145		
≥ 300	5	10	1.27 (0.37–4.45)	0.691		
Smoking	3	9	0.49 (0.14–1.66)	0.248		
Hypertension	4	9	0.98 (0.33–2.91)	0.964		
Diabetes	1	3	0.95 (0.13–7.24)	0.962		
Admission SBP, mmHg	123 (115, 137.5)	125 (114, 130)	1.02 (0.99–1.05)	0.156		
Admission hemoglobin (g/L)	120.1 ± 14.3	124.59 ± 20.82	0.99 (0.97–1.01)	0.450		
PLT, *10^9^/L	167.3 ± 63.5	195.67 ± 55.78	0.99 (0.99–1.00)	0.154		
WBC, *10^9^/L	7.4 ± 3.2	8.30 ± 2.44	0.91 (0.76–1.08)	0.272		
Number of lobes involved	5.0 (2.0, 8.5)	4.0 (2.0, 7.0)	1.07 (0.96–1.19)	0.206		
Bronchial dilatation severity score	2.0 (1.0, 2.9)	1.8 (1.0, 2.6)	1.15 (0.68–1.96)	0.598		
Bronchial wall thickening severity score	2.7 (1.95, 3.0)	2.3 (1.8, 3.0)	1.29 (0.69–2.41)	0.418		
Bronchiectasis type						
Columnar or varicose	8	30	1		**1**	
Cystic	13	9	3.48 (1.44–8.43)	0.006	2.79 (1.12–6.96)	0.028
Embolic materials						
PVA	17	32	1	–		
Embosphere	4	7	1.10 (0.37–3.29)	0.860		
In-hospital days	5.0 (4.0, 7.5)	5.0 (4.0, 9.0)	0.95 (0.83–1.09)	0.438		

Note: Continuous data are presented as the mean ± standard deviation or median (interquartile range). *BAE* Bronchial artery embolization, *HR* Hazard ratio, *CI* Confidence interval, *BMI* Body mass index, *SBP* Systolic blood pressure, *PLT* Blood platelet, *WBC* White blood cell, *PVA* Polyvinyl alcohol

## Discussion

In the present study, we evaluated BAE as a treatment for frequent hemoptysis caused by bronchiectasis. Technical success was achieved in all patients, and instant hemostasis was achieved in the majority of the patients (92.8%). No major procedure-related complications occurred. The 1-, 2-, and 3-year hemoptysis-free survival rates were higher in patients treated with BAE than in patients receiving conservative treatment. Our results demonstrated that BAE yielded a higher hemostasis rate and better long-term control of hemoptysis than conservative treatment for those patients with frequent-onset bronchiectasis.

Frequent hemoptysis is common in patients with bronchiectasis. Recurrent episodes over a long course (despite being non-life-threatening) and the occurrence of frequent hemoptysis can indeed impair the quality of life and cause anxiety in bronchiectasis patients. In clinical practice, hemostatic drugs are ordered initially for patients with mild hemoptysis. However, with the recurrence of each episode of hemoptysis, conservative treatment often results in relatively weak hemoptysis-free control or even disease progression. Our results indicated that BAE achieved better long-term control of hemoptysis than conservative treatment for these frequent-onset bronchiectasis patients. This benefit of BAE was also made evident by a multivariate analysis. As a reliable treatment option, BAE occludes the systemic arterial inflow into the fragile vessels within inflammatory tissue and reduces the perfusion pressure and the likelihood of further bleeding [16]. Although the high control rates of BAE in massive hemoptysis due to bronchiectasis have been well described by previous reports [15, 25, 26], this study initially demonstrated that BAE was also reliable for the management of bronchiectasis-induced frequent hemoptysis. However, the hemoptysis-free rates in the present study were slightly lower than those in previous studies [15, 18]. This might be because the severity of disease in bronchiectasis patients with frequent hemoptysis was more serious. On the other hand, this finding also suggests that after successful hemostasis, these bronchiectasis patients still require long-term comprehensive management, which is also worthy of further investigation.

Furthermore, our results indicated that the cystic type of bronchiectasis was a risk factor for recurrence, even in patients receiving BAE. This may be attributed to the nature of this pathomorphological change. The presence of cystic bronchiectasis facilitates the local accumulation of fluid and bacterial colonization, which causes repeated bacterial infections and continuous inflammation within the bronchial wall and surrounding tissue. These processes stimulate the formation of new blood vessels, which are usually fragile and easily rupture and bleed. Even though BAE may control hemoptysis with a longer duration, the benefit seems to be inferior to the effect accompanying the deterioration of a specific bronchiectasis subtype. Consequently, lobectomy or segmentectomy may be another choice for managing localized cystic lesions in patients with bronchiectasis-related frequent hemoptysis [13, 14].

During the follow-up period, five patients in the conservative treatment group died due to recurrent hemoptysis. These results suggested that BAE might be conducive to reducing mortality in bronchiectasis patients with frequent hemoptysis. Previous studies have indicated that hemoptysis is a main symptom and a trigger of acute exacerbation in bronchiectasis patients, which leads to increased mortality [9, 10]. The longer hemoptysis-free time acquired by receiving BAE may, on the other hand, reduce the likelihood of acute exacerbation. Further research is needed to confirm whether BAE treatment can reduce the frequency of exacerbations in bronchiectasis patients with frequent hemoptysis.

The following limitations of the present study should be mentioned. First, it was a retrospective investigation, with data extracted from a single center. There were differences in baseline characteristics between the BAE and conservative treatment groups. Although multivariate analysis was employed to confirm our conclusion, prospective studies are still warranted to confirm the benefit of BAE for patients with bronchiectasis-related frequent hemoptysis. Second, we evaluated the bronchiectasis severity by the modified Reiff score but did not investigate all clinical indexes, such as the bronchiectasis severity index or FACED scores, which might also be related to recurrent hemoptysis.

## Conclusions

In conclusion, our study provides preliminary evidence that BAE is an alternative, effective and safe treatment for frequent hemoptysis caused by bronchiectasis. The presence of cystic bronchiectasis was independently associated with recurrent hemoptysis, which may raise a concern regarding first-line treatment exploration.

## Supplementary Information

**Additional file 1: Supplementary material 1.** Clinical raw data of the 107 patients with long-term outcomes in the present study.

## Data Availability

All data generated or analysed during this study are included in this published article.

## Abbreviations

BAE: Bronchial arterial embolization
CTA: CT angiography
NBSAs: Non-bronchial systemic collateral arteries
PVA: Polyvinyl alcohol
BMI: Body mass index
SD: Standard deviations
WBC: White blood cell
PLT: Blood platelet
SBP: Systolic blood pressure
HR: Hazard ratio
CI: Confidence interval
IQR: Interquartile range
